# A microarray whole-genome gene expression dataset in a rat model of inflammatory corneal angiogenesis

**DOI:** 10.1038/sdata.2016.103

**Published:** 2016-11-22

**Authors:** Anthony Mukwaya, Jessica M. Lindvall, Maria Xeroudaki, Beatrice Peebo, Zaheer Ali, Anton Lennikov, Lasse Dahl Ejby Jensen, Neil Lagali

**Affiliations:** 1 Department of Ophthalmology, Institute for Clinical and Experimental Medicine, Faculty of Health Sciences, Linkoping University, Linköping 58183, Sweden; 2 National Bioinformatics Infrastructure Sweden (NBIS) Science for Life Laboratory, Department of Biochemistry and Biophysics, Stockholm University, Stockholm S-10691, Sweden; 3 Department of Medical and Health Sciences, Division of Cardiovascular Medicine, Linköping University, Linköping 581 83, Sweden; 4 Department of Microbiology, Tumor and Cell biology, the Karolinska Institute, Stockholm 17177, Sweden

**Keywords:** Experimental models of disease, Inflammation, Microarray analysis

## Abstract

In angiogenesis with concurrent inflammation, many pathways are activated, some linked to VEGF and others largely VEGF-independent. Pathways involving inflammatory mediators, chemokines, and micro-RNAs may play important roles in maintaining a pro-angiogenic environment or mediating angiogenic regression. Here, we describe a gene expression dataset to facilitate exploration of pro-angiogenic, pro-inflammatory, and remodelling/normalization-associated genes during both an active capillary sprouting phase, and in the restoration of an avascular phenotype. The dataset was generated by microarray analysis of the whole transcriptome in a rat model of suture-induced inflammatory corneal neovascularisation. Regions of active capillary sprout growth or regression in the cornea were harvested and total RNA extracted from four biological replicates per group. High quality RNA was obtained for gene expression analysis using microarrays. Fold change of selected genes was validated by qPCR, and protein expression was evaluated by immunohistochemistry. We provide a gene expression dataset that may be re-used to investigate corneal neovascularisation, and may also have implications in other contexts of inflammation-mediated angiogenesis.

## Background & Summary

Angiogenesis drives age-related macular degeneration (AMD), retinopathy of prematurity (ROP)^1^, and diabetic retinopathy (DR), which can also be associated with retinal vein occlusion (RVO)^2^. In the cornea, corneal neovascularisation (CorNV) is driven primarily by inflammation and/or hypoxia^3^. These stimuli can tip the balance between antiangiogenic and proangiogenic molecules, in favour of the latter^4^. In recent years, anti-VEGF therapy has been used to regress immature vessels^5^, but despite the benefits of anti-VEGF therapy, it has been shown to regress new vessels only partially^6,7^, to cause neurotoxicity and in some cases to increase the risk of stroke^8^. In a study focused on CorNV, anti-*VEGFA* therapy was shown to have only a 14% efficacy in regressing neovessels during the early sprouting stages in the rat inflammatory model of angiogenesis^9^. On the other hand, factors other than VEGF have been identified to independently stimulate corneal angiogenesis^10^, while other factors may play a role in vessel maturation. For example, PDGF and Ang1 are reported to be essential for pericyte recruitment to stabilise newly formed vasculature^11,12^, and recently, WNT signalling enhancer R-spondin3 (RSPO3) expressed by ECs has been identified to be crucial for maintaining a remodeling vasculature^13^. The challenge however, is that angiogenesis in a clinical scenario such as corneal transplant rejection^14^, in age-related macular degeneration^15^, and in a tumor microenvironment^16^, occurs in a more complex pro-inflammatory environment, with many players involved at the gene level.

Here, we mimicked pathological angiogenesis using a novel reversible model of suture-induced inflammatory CorNV in the rat, and then studied gene expression profiles associated with angiogenic sprouting and induced blood vessel regression/remodeling. The whole transcriptome was assessed using Affymetrix Rat Gene 2.0 ST Arrays, custom designed for up to 28,407 total RefSeq transcripts coverage. Unlike traditional array designs that rely on probes designed to the first exon of the genes’ 3' end, the Affymetrix Rat Gene 2.0 ST arrays include hundreds of thousands of probes designed to every exon of every transcript represented on the array. The high transcript coverage (median of 22 probes/gene) gives a more accurate detection of genome-wide transcript expression changes compared to traditional microarrays. The whole-transcript analysis approach enables detection of multiple transcript isoforms from a given gene such as splice variants, non-polyadenylated transcripts, transcripts with alternative polyadenylation sites and truncated transcripts. The gene expression for selected genes was validated using qPCR using custom designed primers, and protein expression and localization in the tissue was evaluated by immunohistochemistry. Finally, CEL and CHIP files, and the corresponding sample metadata for each of the samples were deposited in to Gene Expression Omnibus (Data Citation 1).

The dataset described and presented here offers a means to identify and understand transcripts and gene interactions in angiogenesis driven primarily by inflammation. In addition to the coding transcripts, the microarray chips used in this study also capture information about miRNAs, which are reported to be key regulators for biological processes such as the modulation of tip cell behaviour in zebrafish^17^. Also, using the rat alkali burn model, miR-446 among others was suggested to have a role in protecting the transplanted cornea by suppressing its target Prox1 (ref. 18). The provided dataset therefore also provides a means to investigate miRNAs associated with angiogenic sprouting and regression in the cornea. In addition, the data files described here were previously analyzed in-depth, and it was shown that suture removal induced blood vessel remodelling, and that many genes classified as either pro-angiogenic/pro-inflammatory or anti-angiogenic/pro-maturation were identified. At the phenotypic level, blood vessel remodelling was characterized by an increase in macrophages, and a reduction of granulocytes in the cornea^19^.

## Methods

All methods described here are either an extract or an expansion of those recently published, GeneChip WT PLUS Reagent Kit, P/N 703174 Rev. 2 (Affymetrix Inc), and RMA method^19,20^.

### Rats

Ethical application, strain and housing conditions of the animals used for this study were previously detailed^19^. Using 12–16 weeks old rats, the suture model of inflammatory corneal neovascularisation was used as previously described^21^.

### Study design and sample collection

Two 10-0 nylon sutures were placed in the temporal side of the cornea of the right eye of the rat at 1.5 mm from the cornea-scleral border (limbus), to induce inflammation leading to limbal capillary sprouting and CorNV. After 4–5 days following suturing (0 h time point, when new sprouts had invaded the clear cornea one-half to two-thirds of the distance to the suture), rats were divided into two groups, called suture IN and suture OUT. In the suture IN group, sutures were left in place for an additional 24 h before harvesting cornea tissue for gene expression analysis. In the suture OUT group, both sutures were removed at 0 h and corneas were harvested 24 h later for gene expression analysis. The control group consisted of rats whose corneas were not sutured. In both suture IN and suture OUT arms, only the vascularised area of the tissue was excised and immediately immersed in RNA later and stored at −20 °C. For each of the four groups (i.e., Control, 0 h, 24 h suture IN, 24 h suture OUT), *n*=4 rats were used, and the identical phenotype was confirmed by *in vivo* slit lamp and IVCM imaging at 0 h. A detailed graphical illustration of the experimental design we followed was previously published^19^.

### Cornea neovascularisation phenotype

**Figure 1:**
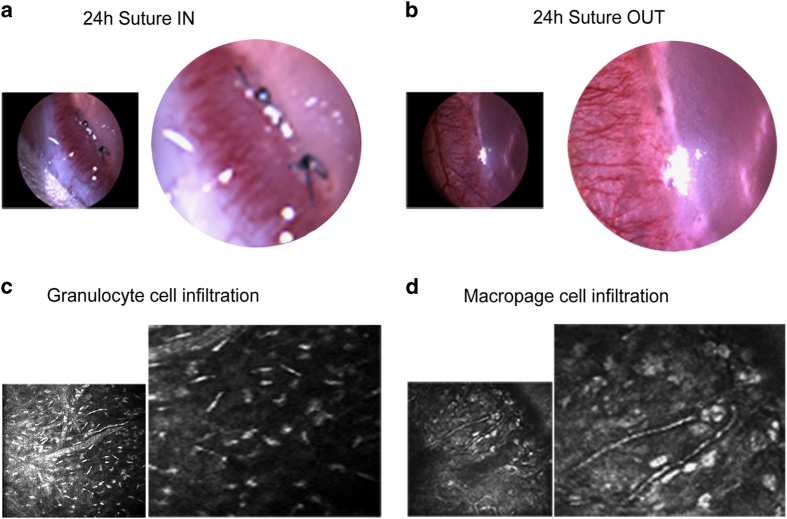
Neovessels and infiltrating inflammatory cells (granulocyte and macrophage) in the sutured cornea. In **a**,**b** are slit lamp images of a neovascularised cornea in the suture IN and suture OUT groups respectively. In **c**,**d** are IVCM images of granulocyte and macrophage cell types infiltrating into the neovascularised cornea, respectively.

Prior to gene expression analysis, morphological data was collected using a clinical slit lamp camera and an *in vivo* confocal microscope (IVCM). The morphological data was analysed for changes in neovessels (for example vessel density and splitting), and for the dynamics of the infiltrating inflammatory cells. From this data, we confirmed the induction of a remodelling phenotype following the removal of the angiogenic stimulus^19^. These findings were of importance as they served as a foundation for the design and planning of the microarray experiments that followed, and whose dataset we now describe here. In Fig. 1a are the slit lamp images of corneas in suture IN and suture OUT groups, indicating new capillary sprouting from the limbus into the cornea. Figure 1b is an example of IVCM images showing two types of inflammatory cells known to infiltrate the sutured cornea, which in this model were affected by removal of the angiogenic stimulus.

### RNA isolation, quantitation and quality verification

**Figure 2:**
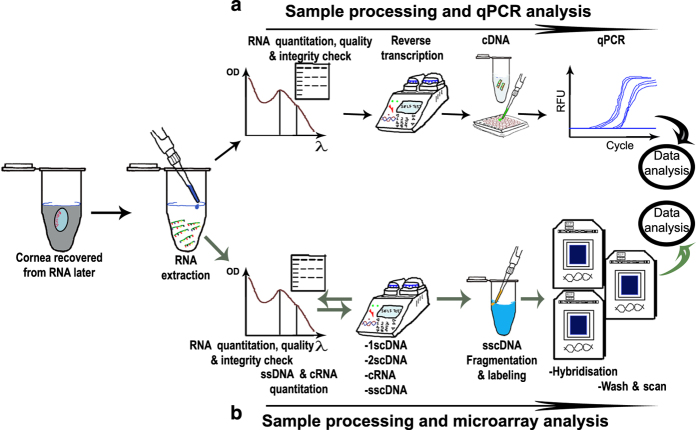
Work flow for RNA extraction and downstream processing for (a) qPCR and (b) microarray analysis. In both cases, cornea tissue previously preserved in RNA later was recovered by thawing at room temperature. The tissue was then disrupted and total RNA extracted from the lysate. The RNA was quantified, purity verified and integrity checked prior to the selected analysis. Separate groups of rats were used for qPCR and microarray analysis. At least three biological replicates were used for each case (i.e., qPCR and microarray analysis). The number of replicates was chosen to increase the statistical reliability of the generated dataset (R^2^>0.96 for samples within a specific sample group).

A single cornea per sample (non-pooled) was used for total RNA extraction. Tissues were preserved in RNA later immediately after harvest and stored at −20 °C. Cornea tissue was disrupted using a hand-held tissueRuptor and disposable Probe (Qiagen, Hilden, Germany), in a 2 ml micro centrifuge tube, on ice. Total RNA was then extracted from the lysate (Qiagen, Hilden, Germany), without DNase treatment. RNA concentration was then determined using a Nanodrop spectrophotometer 2000 (Thermo scientific), and the RNA quality verified using an Agilent 2100 Bioanalyzer (Agilent RNA 6000 Nano Kit Quick Start guide, Agilent Technologies). RNA Integrity Number (RIN)≥7 was set as the cut off for selecting RNA samples for downstream analysis. Samples with RIN values less than the set cut-off were excluded from the analysis process, and were replaced with fresh sample that met the inclusion criteria. Speed vac was used to concentrate samples whenever needed. RNA samples were temporarily stored at −20 °C following extraction and for both microarray and qPCR analysis, the RNA was used for analysis not longer than 7 days’ post extraction. See Fig. 2a for an illustration of the workflow that was followed during sample processing and qPCR analysis.

### Microarray target preparation, hybridisation and data acquisition

All procedures for microarray analysis were performed at the core facility, cell biology laboratory at Linköping University. Gene expression analysis was performed using the whole-transcriptome Rat Gene 2.0 ST microarrays (Affymetrix Inc). An input of 100 ng of total RNA was mixed with the Poly-A RNA Spike-in controls and used for microarray target preparation following the manufacturer’s instructions (GeneChip WT PLUS Reagent Kit, P/N 703174 Rev. 2, Affymetrix Inc). Fifteen Rat Gene 2.0 ST arrays were used to correspond to 4 microarray chips per experimental time point (except for the 0 h, where 3 microarrays were used as one as one sample was lost (spillage) during target processing). In summary; single-stranded cDNA with T7 promoter sequence at the 5' end was synthesised from total RNA using primers containing a T7 promoter sequence. Following DNA polymerase and RNase H, double-stranded cDNA was synthesised from the single-stranded cDNA. After this, cRNA was synthesized and amplified by *in vitro* transcription (IVT) of the second-stranded cDNA template using T7 RNA polymerase. The cRNA was purified and the yield verified using Nanodrop 2000. Reverse transcription of cRNA using 2nd cycle primers synthesised 2nd-Cycle Single-Stranded cDNA. In order to receive single-stranded cDNA, the 2nd-Cycle Single-Stranded cDNA was treated with RNase H to hydrolyse cRNA template, which was purified and quantified as already described above. Following fragmentation of the Single-stranded cDNA using uracil-DNA glycosylase (UDG) and apurinic/apyrimidinic endonuclease 1 (APE 1) at the unnatural dUTP residues, the fragmented cDNA was labeled by terminal deoxynucleotidyl transferase (TdT) using the Affymetrix proprietary DNA Labeling Reagent that was covalently linked to biotin. The labeled cDNA was then hybridised to the Affymetrix Rat Gene 2.0 ST arrays in a 645 hybridisation oven and washed in Fluidics Station 450, according to the User's Guide (AGCC, P/N 08–0295, Affymetrix Inc). The chips were then scanned according to the user Manual for Cartridge Arrays (PN 702731, Affymetrix Inc). The acquired image files were examined for hybridisation of the internal Poly-A RNA Spike-in controls. DAT and CEL files were then generated from the probe intensity files using GeneChip Command Console Software (AGCC). See Fig. 2b for an illustration of the workflow that was followed during sample and microarray preparation and analysis.

### Analysis of microarray files

Raw CEL files were converted into expression measures, background-corrected, and data-normalized using Affymetrix Expression Console software and Affymetrix transcription console (TCA) v. 3.0 tool.

**Figure 3:**
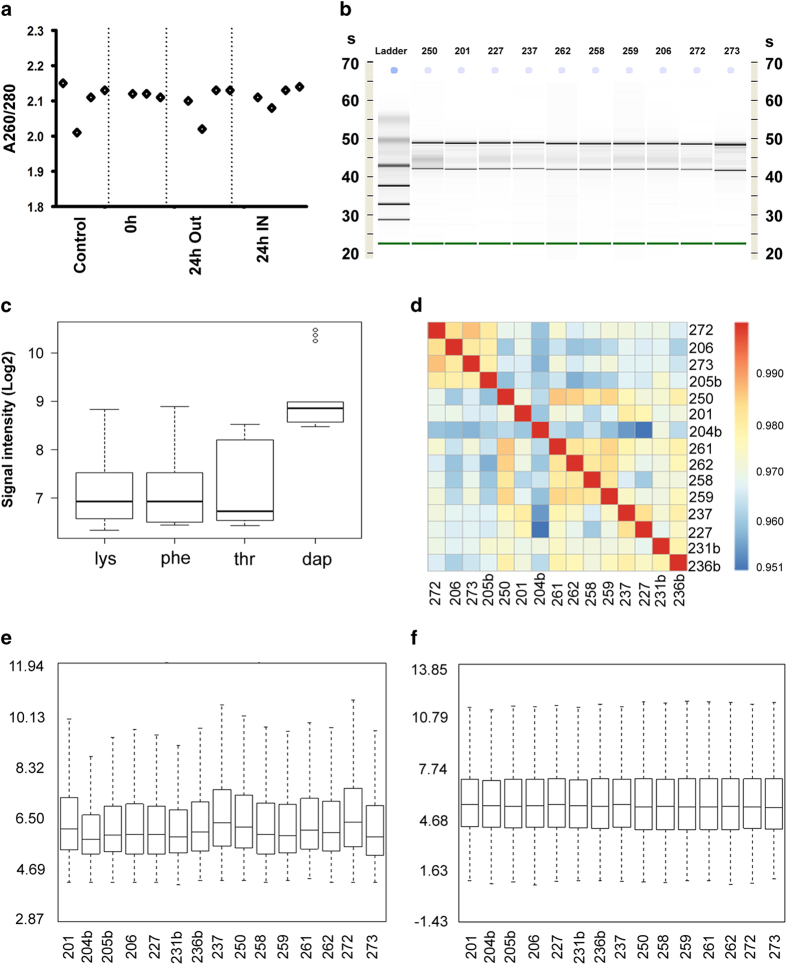
High quality RNA was extracted from a single cornea, and was sufficient for whole transcriptome analysis. All CHIPs passed the initial quality checks. (**a**) RNA purity as determined by Nanodrop 2000, (**b**) is the gel size separation of the 18S and 28S subunits shown as distinct bands, and S, integration number. Results in **b** were obtained using RNA 6000 pico kit following the eukaryote total RNA Pico assay. (**c**) Boxplot of four of Affymetrix Spike-In controls, lys-M, phe-M, thr-M and dap-M illustrating the range of signal (log2) over the experiment as well as quality control of the arrays technical run. (**d**) Illustrates the Pearsson Correlation for all samples after DABG correction and RMA normalization (figure constructed using R/Bioconducter and package pheatmap). (**e**,**f**) Boxplot showing raw log_2_ Probe Cell Intensities, before (**e**) and after the DABG correction and RMA normalized log_2_ Expression Signal (**f**) for all samples.

The Poly-A RNA Spike-in controls that were previously mixed with the pure and intact RNA sample Figure 3a,b, were successfully detected on the microarray chips Fig. 3c. Pearson’s Correlations showed high sample correlation with sample groups, with a higher correlation found between the control samples Fig. 3d. Also, the control samples are separate from the experimental samples. Robust Multi-array Average (RMA) for background correction, normalisation and computation of probe-set level expression of the samples was successful Fig. 3e,f.

Processing and normalization of the raw-files, and CEL files was done using the Affymetrix Expression Console built in algorithm called ‘Detection above background’ (DABG) and the RMA method, which uses a multi-array approach in its normalization method. The DABG algorithm is intended to serve as a confidence score in lieu of Affymetrix previous Detection call (‘Present’ or ‘Absent’). In short, DABG is a detection metric generated by comparing ‘Perfect Match’ probes to a distribution of background probes. This comparison yields a *P*-value which is then combined into a probe set level *P*-value using the Fischer equation. More information regarding the DABG algorithm is found at: http://media.affymetrix.com/support/technical/whitepapers/exon_background_correction_whitepaper.pdf. The RMA package includes background correction, normalization and computation of probe-set level expression. The data was then log_2_ transformed for further biological downstream analysis. Comprehensive literature about the RMA method is available elsewhere^20^.

### Verification of fold change expression by quantitative real-time PCR (qPCR)

**Figure 4:**
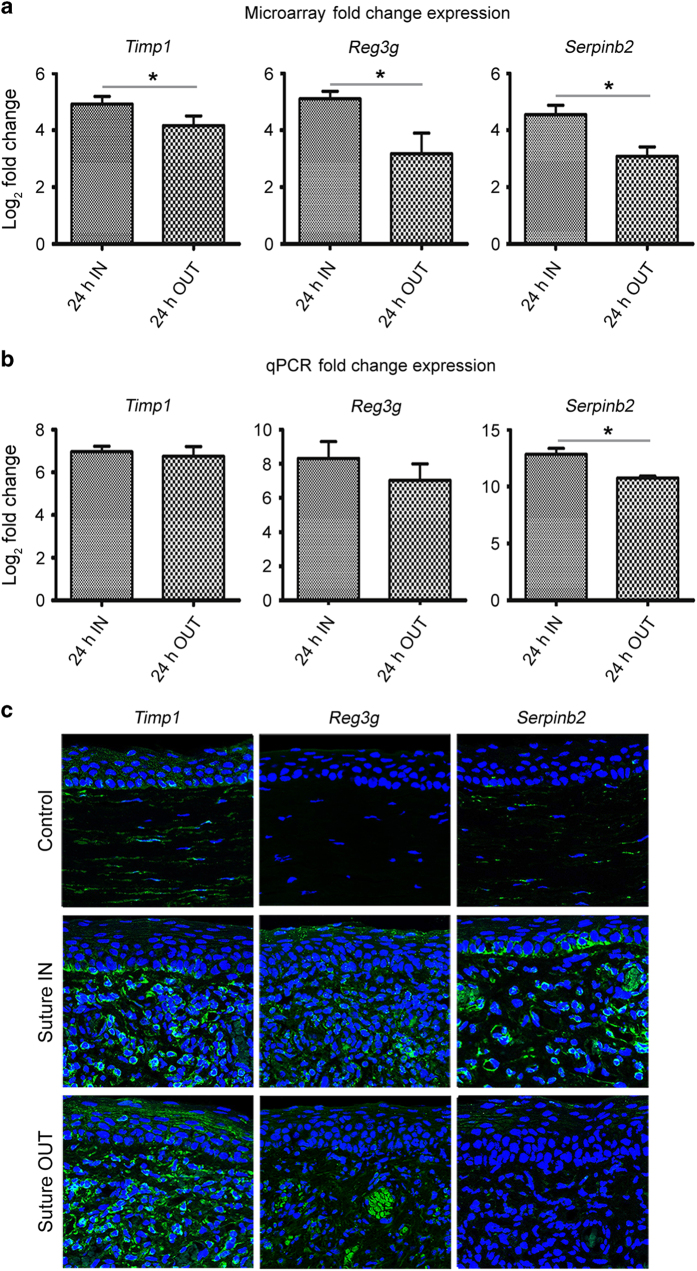
Validation of fold change expression by qPCR and investigation of protein expression and localisation at the tissue level by immunohistochemistry. (**a**) is the microarray log_2_ fold change expression, while (**b**) is the corresponding qPCR log_2_ fold change expression. *n*=3 and 4, for (**a**,**b**) respectively. For comparison of the log_2_ fold change between suture IN and suture OUT, an unpaired two-tailed *t*-test was performed to compare the means between suture IN and suture OUT. No multiple test correction was performed for the two-group comparisons. The *t* test was performed in both the microarray and qPCR datasets independently. In both (**a**,**b**), asterisks indicate significance for *P*<0.05 level, error bars represent standard deviation. Data was analysed using GraphPad Prism 5 (GraphPad Software, Inc. CA 92037 USA). In (**c**) are immunofluorescent images from cornea sections indicating the corresponding protein expression across treatments. DAPI labels cell nuclei in blue, while the target proteins are indicated in green color.

A separate group of rats was used for this purpose. Total RNA was extracted as illustrated in Fig. 2a above. From the RNA, cDNA was synthesised at 42 °C for 2 h with a heated lid ON (Invitrogen). Custom Taqman primers were obtained from TaqMan, Applied Biosystems for *Timp1*, *Serpinb2* and *Reg3g*. The primers were used with TaqMan Fast Advanced Master Mix (Applied Biosystems). Three biological, and 2 technical replicates were used per sample and the analysis was performed using CFX 96 Touch Real-Time PCR detection system (Bio-Rad). Threshold cycle (Ct) values were normalised to GAPDH, and gene expression was measured by the relative quantitation method (2^−ΔΔCt^). The fold change expression profile between suture IN and suture OUT as determined by qPCR in general was in agreement with the expression profile determined by the microarray analysis Fig. 4a,b, with further qPCR results available^19^.

### Protein expression and localisation by immunohistochemistry

Following qPCR confirmation of the fold change, protein expression and localisation in cornea tissue cross-sections was investigated. A separate set of three rats per group was used for this purpose. Immunohistochemistry was performed to assess the expression and localisation for *Timp1*, *Serpinb2* and *Reg3g*, using primary polyclonal antibodies; orb195994 and orb25969 from Biorbyt for *Timp1* and *Reg3g* respectively, and ABIN1860122 from antibodiesonline.com, for *Serpinb2*. For detection, Alexa 488 secondary fluorescent antibody was used. From the analysis, *Timp1* was expressed and localised within the stroma and endothelium, *Reg3g* localised to the stroma and *Serpinb2* to the epithelium, stroma and endothelium Fig. 4. The protein expression of several further genes in the corneal tissue has been provided elsewhere^19^.

## Data Records

CEL and CHIP files associated with the samples analysed in this study are deposited at gene expression omnibus with the accession number GSE81418. The detailed information regarding the samples is tabulated in Table 1.

## Technical Validation

A number of precautions were taken into consideration during the experimental procedure. Rats were randomly assigned to experimental groups (suture IN and suture OUT) at 0 h. The rat ID assigned to the rats at the beginning of each experiment was maintained throughout the entire experimental process, and this included both microarray and qPCR validation of gene expression data. Following rat sacrifice, cornea tissue was harvested from the eye within one minute, and immediately immersed in RNA later and stored at −20 °C. This practice was instated to minimize alterations in gene expression that could arise from cold ischemia^22^. Surgical equipment were cleaned between tissue extraction from different animals, and after each experimental day. At each time point, four biological replicates were used. RNA was extracted from individual rats without pooling of any samples. Only high quality RNA (refer to the RIN values) was used for both microarray and qPCR analysis.

## Usage Notes

Microarrays have been used previously with great success as a tool for an unbiased identification of novel pathways and regulatory molecules in the pathology of different diseases^23,24^. These datasets therefore provide a solid basis for the investigation of specific research questions of interest e.g. angiogenic sprouting, remodeling of capillaries, relationship between inflammatory cells and capillaries, and hypoxia among others. The microarray chips used in this study also provide information regarding microRNAs that are key regulators of gene expression. Using this dataset, the role of these molecules can be explored to address questions regarding the functionality of both known and novel gene targets that are key in inflammation-driven angiogenesis in the cornea. To explore such possibilities, different Gene Set Expression Analysis (GSEA) software can be used. These are available both as public tools such as STRING and Cytoscape with for instance the Bingo or ClueGO, and also as commercially available software such as QIAGEN's Ingenuity Pathway Analysis tool. Information pertaining miRNAs can also be exploited by using specific kits designed for miRNA extraction and for qPCR validation of gene expression. These kits are commercial and readily available.

Probe IDs with missing gene symbols can be updated by performing a Batch Query at NetAffx Analysis Center tool-Affymetrix. Gene fold change expression can be obtained by comparing each treatment group to the non-sutured control sample group. The corresponding p and q values can also be calculated since the study has enough degrees of freedom in its statistical design. For the adjusted *P*-value calculation (False Discovery Rate (FDR) or q value) researchers commonly use the Benjamini-Hochberg statistical method. However, other methods exist and are used. The method of choice is dependent on the study design.

## Additional Information

**How to cite this article:** Mukwaya, A. *et al.* A microarray whole-genome gene expression dataset in a rat model of inflammatory corneal angiogenesis. *Sci. Data* 3:160103 doi: 10.1038/sdata.2016.103 (2016).

**Publisher’s note:** Springer Nature remains neutral with regard to jurisdictional claims in published maps and institutional affiliations.

## Supplementary Material



## Figures and Tables

**Table 1:** Sample description.

**ID**	**GEO-ID**	**Organism**	**Age (weeks)**	**Tissue**	**Sample description**	**Analysis**
206	GSM2152148	Rattus norvegicus albino	16	Cornea	Control, non-sutured	Microarray
205b	GSM2152149	Rattus norvegicus albino	16	Cornea	Control, non-sutured	Microarray
272	GSM2152150	Rattus norvegicus albino	16	Cornea	Control, non-sutured	Microarray
273	GSM2152151	Rattus norvegicus albino	16	Cornea	Control, non-sutured	Microarray
204b	GSM2152152	Rattus norvegicus albino	16	Cornea	Sutured cornea, 0 h	Microarray
201	GSM2152153	Rattus norvegicus albino	16	Cornea	Sutured cornea, 0 h	Microarray
250	GSM2152154	Rattus norvegicus albino	16	Cornea	Sutured cornea, 0 h	Microarray
227	GSM2152155	Rattus norvegicus albino	12	Cornea	24 h Suture OUT	Microarray
231b	GSM2152156	Rattus norvegicus albino	12	Cornea	24 h Suture OUT	Microarray
236b	GSM2152157	Rattus norvegicus albino	12	Cornea	24 h Suture OUT	Microarray
237	GSM2152158	Rattus norvegicus albino	12	Cornea	24 h Suture OUT	Microarray
262	GSM2152159	Rattus norvegicus albino	13	Cornea	24 h Suture IN	Microarray
258	GSM2152160	Rattus norvegicus albino	13	Cornea	24 h Suture IN	Microarray
259	GSM2152161	Rattus norvegicus albino	13	Cornea	24 h Suture IN	Microarray
261	GSM2152162	Rattus norvegicus albino	13	Cornea	24 h Suture IN	Microarray
